# Associations between insurance-related affordable care act policy changes with HPV vaccine completion

**DOI:** 10.1186/s12889-021-10328-4

**Published:** 2021-02-06

**Authors:** Summer Sherburne Hawkins, Krisztina Horvath, Jessica Cohen, Lydia E. Pace, Christopher F. Baum

**Affiliations:** 1 Boston College, School of Social Work, McGuinn Hall, 140 Commonwealth Avenue, Chestnut Hill, MA 02467 USA; 2 Boston College, Department of Economics, Chestnut Hill, MA USA; 3 Harvard T.H. Chan School of Public Health, Department of Global Health and Population, Boston, MA USA; 4 Brigham and Women’s Hospital, Boston, MA USA; 5 German Institute for Economic Research (DIW Berlin), Department of Macroeconomics, Berlin, Germany

**Keywords:** Human papillomavirus, Vaccine, Affordable care act, Policy, Health disparity

## Abstract

**Background:**

Although all 11- or 12-year-olds in the US were recommended to receive a 3-dose series of the human papillomavirus (HPV) vaccine within a 12-month period prior to 2016, rates of completion of the HPV vaccine series remained suboptimal. The effects of the Affordable Care Act (ACA), including private insurance coverage with no cost-sharing and health insurance expansions, on HPV vaccine completion are largely unknown. The aim of this study was to examine the associations between the ACA’s 2010 provisions and 2014 insurance expansions with HPV vaccine completion by sex and health insurance type.

**Methods:**

Using 2009–2015 public and private health insurance claims from Maine, New Hampshire, and Massachusetts, we identified 9-to-26-year-olds who had at least one HPV vaccine dose. We conducted a logistic regression model to examine the associations between the ACA policy changes with HPV vaccine completion (defined as receiving a 3-dose series within 12 months from the date of initiation) as well as interactions by sex and health insurance type.

**Results:**

Over the study period, among females and males who initiated the HPV vaccine, 27.6 and 28.0%, respectively, completed the series within 12 months. Among females, the 2010 ACA provision was associated with a 4.3 percentage point increases in HPV vaccine completion for the privately-insured (0.043; 95% CI: 0.036–0.061) and a 5.7 percentage point increase for Medicaid enrollees (0.057; 95% CI: 0.032–0.081). The 2014 health insurance expansions were associated with a 9.4 percentage point increase in vaccine completion for females with private insurance (0.094; 95% CI: 0.082–0.107) and a 8.5 percentage point increase for Medicaid enrollees (0.085; 95% CI: 0.068–0.102). Among males, the 2014 ACA reforms were associated with a 5.1 percentage point increase in HPV vaccine completion for the privately-insured (0.051; 95% CI: 0.039–0.063) and a 3.4 percentage point increase for Medicaid enrollees (0.034; 95% CI: 0.017–0.050). In a sensitivity analysis, findings were similar with HPV vaccine completion within 18 months.

**Conclusions:**

Despite low HPV vaccine completion overall, both sets of ACA provisions were associated with increases in completion among females and males. Our results suggest that expanding Medicaid across the remaining states could increase HPV vaccine completion among publicly-insured youth and prevent HPV-related cancers.

**Supplementary Information:**

The online version contains supplementary material available at 10.1186/s12889-021-10328-4.

## Background

In the US, an estimated 34,800 cancers were attributable to the human papillomavirus (HPV) annually during 2012–2016, with cervical and oropharyngeal cancers being the most common [1]. Despite the HPV vaccine being one of the most effective measures to prevent the majority of cervical and other HPV-related cancers [1, 2], completion of the multi-dose series remains suboptimal in the US. The Advisory Committee on Immunization Practices (ACIP) has recommended that females receive the HPV vaccine since 2006 [3] and males since 2011 [4]. ACIP initially recommended that all 11- or 12-year-olds receive a 3-dose series within a 12-month period until age 26 years if not vaccinated previously for females and until age 21 for males [2, 5]. Revised guidelines in December 2016 permit a 2-dose series for girls and boys who receive their first HPV vaccine at ages 9–14 years, while still requiring a 3-dose series for older or immunocompromised adolescents [6]. In 2017, the National Immunization Survey–Teen (NIS-Teen) showed that while 68.6% of 13–17-year-old females initiated the HPV vaccine series, only 53.1% completed the recommended sequence; however, corresponding figures for males were 62.6 and 44.3%, respectively [7]. Increasing the proportion of female and male adolescents who complete the HPV vaccine series is a national priority (Healthy People 2020 IID:11.4 and IID-11.5) [8].

The Patient Protection and Affordable Care Act (ACA) rolled out a series of provisions that removed barriers to accessing the HPV vaccine. In September 2010, the ACA required non-grandfathered private plans to cover the HPV vaccine with no patient cost-sharing [9], which removed a significant financial barrier to vaccine uptake [10–12]. At that time, the dependent care provision also came into effect allowing young adults up to age 26 years to remain on their parents’ private health insurance plans. From January 2014, state and federal Marketplaces offering publicly-subsidized private health insurance plans and insurance companies were banned from denying coverage or charging higher premiums for pre-existing conditions. Also in 2014, newly eligible Medicaid enrollees acquired coverage for the HPV vaccine with ACA’s Medicaid expansion [13]. As a result of these ACA provisions, over 19 million more people have acquired health insurance coverage [14], reducing coverage barriers to HPV vaccine uptake.

Evaluations of the ACA have only examined the effects of the 2010 provisions on HPV vaccination among females. Using self-reported data from national surveys, both studies found that the ACA increased HPV vaccine initiation and completion [15, 16]. However, neither assessed differential effects by health insurance status. Furthermore, we are not aware of any studies that have evaluated the ACA’s 2014 insurance-related provisions on HPV vaccine completion among females or males across insurance types. We took a three-pronged approach to address limitations in the current evidence base: first, by using population-level datasets over the time frame that both ACA provisions came into effect; second, by evaluating differences by sex based on changes in clinical recommendations; and third, by assessing heterogeneous effects of both ACA provisions between Medicaid enrollees and the privately-insured. The aim of this study was to examine the associations between the ACA’s 2010 provisions and 2014 health insurance expansions with HPV vaccine completion by sex and insurance type.

## Methods

We used All Payer Claims Databases (APCD) from Massachusetts (MA; Center for Health Information and Analysis), Maine (ME; Maine Health Data Organization) and New Hampshire (NH; Comprehensive Health Care Information System), which collect health insurance claims from insurance companies operating in a given state. APCDs are mandated by law in these states, which ensures that most of the privately- and publicly- (Medicare, Medicaid) insured populations are included. While the standardized data submission requirements allowed us to pool data from the three states, the length of the study sample differed: MA provided data from January 2011 through December 2015, and ME and NH provided data from January 2009 through December 2015. For NH there were no public claims available from April 2013 through November 2013; personal communication with Rose Hess on April 17, 2018. Data are accessible to researchers upon application and costs vary by state.

Our analytic sample included children and young adults aged 9 to 26 years who had at least one HPV vaccine dose during the study period based on claims associated with Current Procedural Terminology codes 90649 (Gardasil), 90650 (Cervarix) and 90651 (Gardasil 9). We restricted the study period to September 2009 through June 2014 (from September 2011 in MA). By excluding 8 months from the beginning and 18 months from the end of the study period, we could ensure the full time period allocated for series completion at either end of data collection. Due to low HPV vaccine initiation prior to the ACIP recommendation for males, we excluded males before October 2011 [17, 18].

Among the 385,998 youth who received at least one dose of HPV vaccine over the study period, we excluded 2701 individuals who received more than 3 doses because we were unable to observationally distinguish data submission errors from intentional repetitions. This resulted in a total sample size of 383,297 9–26-year-olds, with 194,407 females and 188,890 males. The Boston College Institutional Review Board reviewed this study and considered it exempt.

As the HPV vaccination recommendation over the study period consisted of a series of three shots [2, 5],^2, 5^ we constructed an individual-specific completion measure by aggregating all HPV claims received for a given person. Our primary outcome variable was a binary indicator of HPV vaccine completion, defined as receiving a 3-dose series within 12 months from the date of initiation [17].

We examined the associations between two sets of ACA policy changes with HPV vaccine completion during the study period. First, the introduction of the ACA in September 2010 resulted in facilitated dependent care coverage and HPV vaccination without cost-sharing [9]. The elimination of cost-sharing allows young people enrolled in non-grandfathered private insurance plans to receive the HPV vaccine without any copayment, coinsurance or deductible payments. Second, three major provisions of the ACA came into effect in January 2014 that substantially extended access to health insurance coverage and could influence HPV completion. The first provision included Medicaid expansion in MA (January 1, 2014) and NH (August 15, 2014), but not ME; Medicaid expansion plans were eligible to receive the HPV vaccine with no cost-sharing, while coverage varied by state for traditional Medicaid plans [19]. The second provision was the introduction of the health insurance Marketplaces as standardized platforms to purchase publicly-subsidized private health insurance coverage. The third provision was the ban of insurers’ rating practices based on pre-existing conditions. We created binary indicator variables for the policy changes in 2010 and 2014.

We used demographic and insurer information available in the APCD medical claims files to generate covariates for our analyses: sex (male, female), age groups (9–13, 14–18, 19–26 years) at the initiation of the HPV vaccine, state (MA, ME, NH), insurance type (private, Medicaid), and the year of initiation. There were no missing data for covariates as health insurers were required to submit this information to the APCDs. Additional participant socio-demographic information, including race/ethnicity, is not consistently recorded in the APCD.

### Statistical analysis

We calculated descriptive statistics for the analytic sample stratified by the different covariates of interest. We calculated the prevalence of HPV completion by the ratio of those who completed the series based on each definition and those who initiated the vaccine during the study period, i.e. those who received at least one dose of the HPV vaccine. We first examined the prevalence of HPV vaccine completion within 12 months across the demographic and insurance-related characteristics available in the medical claims data stratified by sex. We then estimated unadjusted and adjusted logistic regression models stratified by sex to examine the predictors of HPV vaccine completion. Covariates included age and insurance type, which have been shown previously to be associated with HPV vaccine completion, as well as year and state to account for time trends and regional differences, respectively [7, 15–17]. We also present average completion of the HPV vaccine series stratified by state, sex, and insurance type across each year of the study period.

Next, we conducted an individual-level regression analysis to examine the associations between the insurance-related ACA policy changes with HPV vaccine completion within 12 months. Since our analytic sample consisted of multiple HPV claims for most individuals, we collapsed our dataset to a single observation per person that contained information of the characteristics of the entire series of HPV claims (total number of doses, date of initiation, number of months between each dose, months between the first and last doses). We estimated logistic regression models where we modeled the probability of series completion as a function of the ACA policy breaks and demographic characteristics. For the dependent variable, we used a binary indicator which takes the value one if our completion definition is satisfied, zero otherwise. In our first, simplest specification, we ran stratified models by sex to identify any heterogeneous policy effects across males and females. Then, we constructed a combined model to directly compare our estimates across these groups. In the final specification we included three-way interaction terms for each policy break by sex and insurance type (males were excluded from the sample at the time of the 2010 ACA break). The model also controlled for the participant’s age group, state and year fixed effects. The final estimates were obtained from the following logistic regression specification:

$$ \Pr \left(\mathrm{completion}\right)=F\left[\sum \limits_{j=2010}^{2014}{\beta}_j{YFS}_i+{\beta}_{ME}{ME}_i+{\beta}_{NH}{NH}_i+{\beta}_{A1518}{AGE}_{1518}+{\beta}_{1926}{AGE}_{1926}+{\beta}_M{M}_i+{\beta}_{PVT}{PVT}_i+{\beta}_{MP}\left({M}_i\times {PVT}_i\right)+{\beta}_{2010} ACA{2010}_i+{\gamma}_i\left({PVT}_i\times ACA{2010}_i\right)+{\beta}_{2014} ACA{2014}_i+{\gamma}_2\left({PVT}_i\times ACA{2014}_i\right)+{\gamma}_3\left({M}_i\times ACA{2014}_i\right)+{\gamma}_4\left({M}_i\times {PVT}_i\times ACA{2014}_i\right)\ \right] $$

where *F*[^.^] is the cumulative distribution function of the logistic distribution, *YFS*_*i*_ denotes year of first shot, *ME* and *NH* refer to states (*MA* omitted), *AGE1518*_*i*_ and *AGE1926*_*i*_ refer to age categories (ages 9–14 omitted), *M*_*i*_ refers to gender male and *PVT*_*i*_ refers to private insurance (Medicaid omitted). *ACA2010*_*i*_ and *ACA2014*_*i*_ are indicators of the ACA policy changes in 2010 and 2014, respectively.

We then conducted two sensitivity analyses in which we repeated this series of analyses by first, extending the definition of our vaccine completion measure and second, reducing the dose schedule. The former outcome was defined as completion of the 3-dose series within 18 months from the date of initiation [17]. In response to the revised 2016 HPV guidelines [6], the latter outcome was defined as completion of the 2-dose series within 12 months from the date of initiation [17].

We report average marginal effects which compute the partial effects of each explanatory variable, i.e. implementation of each ACA policy, on the probability that the observed dependent variable, Y_i_ = 1, i.e. the HPV series was completed, while holding other covariates constant. We calculated differential responses by sex and insurance type as our stratified analysis revealed significant heterogeneity among these groups. Finally, we calculated predictive margins and used an adjusted Wald test to assess the statistical significance of differences by sex and insurance type. We conducted analyses using Stata statistical software version 15.1 (StataCorp, College Station, TX).

## Results

Overall, among females and males who initiated the HPV vaccine over the study period, 27.6% (53,591/194,407) and 28.0% (52,892/188,890), respectively, completed the series within 12 months. Among those who completed the HPV vaccine series, the mean difference in time between the first and last HPV doses was 6.2 months (standard deviation 3.1 months). The prevalence of HPV completion was higher among females and males who initiated the series between ages 9 and 14 compared with those who initiated at older ages and privately-insured youth were more likely to complete the series than Medicaid enrollees (Table 1). In addition, a higher proportion of youth in MA completed the HPV vaccine series than in ME or NH. Associations persisted in adjusted models and were consistent for females and males. The prevalence of vaccine completion also fluctuated significantly across the study period. In adjusted models, vaccine completion in 2013–2014 was significantly lower than the baseline year for females and males. Supplemental Table 1 illustrates these trends in completion of the HPV vaccine series for females and males across insurance type in all three states over the study period.

**Table 1:** Descriptive statistics for HPV vaccine completion defined as completion of the 3-dose series within 12 months

	Females					Males				
	N	%	% series completed	Unadjusted OR (95% CI)	Adjusted^a^ OR (95% CI)	N	%	% series completed	Unadjusted OR (95% CI)	Adjusted^a^ OR (95% CI)
Age of first dose (years)										
9–14	96,702	49.7	30.8	1	1	72,071	38.2	29.7	1	1
15–18	56,407	29.0	26.8	0.83 (0.81–0.84)	0.79 (0.77–0.81)	87,336	46.2	28.6	0.95 (0.93–0.97)	0.86 (0.85–0.88)
19–26	41,298	21.2	21.1	0.60 (0.59–0.62)	0.58 (0.56–0.59)	29,483	15.6	21.9	0.66 (0.64–0.68)	0.60 (0.58–0.62)
Insurance type										
Medicaid	28,827	14.8	17.6	1	1	22,588	12.0	17.8	1	1
Private	165,580	85.2	29.3	1.94 (1.88–2.01)	2.60 (2.08–3.24)	166,302	88.0	29.4	1.93 (1.88–2.00)	1.93 (1.86–2.01)
Year of first dose										
2009^b^	3565	1.8	21.7	1	1	–	–	–	–	–
2010	11,397	5.9	28.2	1.42 (1.29–1.55)	1.42 (1.29–1.56)	–	–	–	–	–
2011	28,463	14.6	26.8	1.32 (1.22–1.44)	0.94 (0.83–1.07)	6484	3.4	31.8	1	1
2012	59,371	30.5	32.6	1.74 (1.61–1.89)	1.14 (1.01–1.30)	74,231	39.3	34.8	1.15 (1.09–1.21)	1.09 (1.04–1.16)
2013	64,134	33.0	22.2	1.03 (0.95–1.21)	0.62 (0.55–0.70)	78,172	41.4	21.4	0.58 (0.55–0.62)	0.52 (0.49–0.55)
2014^b^	27,477	14.1	30.5	1.59 (1.46–1.72)	0.75 (0.65–0.86)	30,003	15.9	27.5	0.81 (0.77–0.86)	0.60 (0.55–0.65)
State										
Massachusetts	136,351	70.1	29.2	1	1	151,877	80.4	29.5	1	1
Maine	24,761	12.7	24.4	0.78 (0.76–0.81)	0.76 (0.73–0.78)	15,160	8.0	19.3	0.57 (0.55–0.60)	0.59 (0.56–0.61)
New Hampshire	33,295	17.1	23.2	0.73 (0.71–0.75)	0.67 (0.65–0.69)	21,853	11.6	23.6	0.74 (0.71–0.76)	0.72 (0.70–0.75)

Abbreviations: CI, confidence interval; HPV, human papillomavirus; OR, odds ratio

^a^ Models were adjusted for all variables

^b^ Partial study year

Among females, the introduction of the ACA in 2010 was associated with a 4.3 percentage point increase in HPV vaccine completion for the privately-insured (0.043; 95% confidence interval [CI]: 0.036–0.061) and a 5.7 percentage point increase for Medicaid enrollees (0.057; 95% CI: 0.032–0.081) (Table 2). Similarly, the 2014 health insurance expansions were associated with a 9.4 percentage point increase in vaccine completion for females with private insurance (0.094; 95% CI: 0.082–0.107) and a 8.5 percentage point increase for Medicaid enrollees (0.085; 95% CI: 0.068–0.102). Across both insurance-related ACA policy changes, there were no differences in vaccine completion for females by insurance type (*p* = 0.3 and p = 0.3, respectively).

**Table 2:** Marginal effects of the associations between insurance-related ACA policy changes and HPV vaccine completion by sex and insurance type

	ACA no cost sharing and dependent care provisions in 2010			ACA insurance reforms in 2014		
	Marginal effect (95% CI)	*P*-value	Wald test P-value^e^	Marginal effect (95% CI)	P-value	Wald test P-value^e^
3-dose HPV series completion within 12 months^a,b^						
Male						0.05
Private insurance	–			0.051 (0.039–0.063)	< 0.01	
Medicaid	–			0.034 (0.017–0.050)	< 0.01	
Female			0.3			0.3
Private insurance	0.043 (0.036–0.061)	< 0.01		0.094 (0.082–0.107)	< 0.01	
Medicaid	0.057 (0.032–0.081)	< 0.01		0.085 (0.068–0.102)	< 0.01	
3-dose HPV series completion within 18 months^a,c^						
Male						0.03
Private insurance	–			0.060 (0.048–0.071)	< 0.01	
Medicaid	–			0.040 (0.022–0.058)	< 0.01	
Female			0.02			0.35
Private insurance	0.033 (0.014–0.052)	< 0.01		0.109 (0.097–0.121)	< 0.01	
Medicaid	0.069 (0.040–0.098)	< 0.01		0.100 (0.082–0.118)	< 0.01	
2-dose HPV series completion within 12 months^a,d^						
Male						< 0.01
Private insurance	–			0.018 (0.006–0.029)	< 0.01	
Medicaid	–			−0.026 (−0.046- -0.006)	0.01	
Female			0.04			0.3
Private insurance	0.094 (0.055–0.134)	< 0.01		0.044 (0.032–0.056)	< 0.01	
Medicaid	0.025 (0.005–0.046)	0.02		0.035 (0.015–0.054)	< 0.01	

Abbreviations: ACA, Affordable Care Act; CI, confidence interval; HPV, human papillomavirus

^a^ Models includes interaction between policy breaks, sex, and insurance type; adjusted for age group, state and year fixed effects

^b^ Recommendation (as of 2015) for females and males aged 9–26 years to receive 3-dose HPV series within 12 months after first dose

^c^ Sensitivity analysis based on recommendation (as of 2015) to extend window of series completion to 18 months after first dose

^d^ Sensitivity analysis based on 2016 recommendation to receive 2-dose HPV series within 12 months after first dose

^e^ *P*-values of Wald tests for the equivalence of predictive margins across insurance types

Among males, the 2014 ACA reforms were associated with a 5.1 percentage point increase in HPV vaccine completion for the privately-insured (0.051; 95% CI: 0.039–0.063) and a 3.4 percentage point increase for Medicaid enrollees (0.034; 95% CI: 0.017–0.050) (Table 2). Although the differences in vaccine completion among males by insurance type were minimal (*p* = 0.05), the 2014 insurance reform had a larger effect among both privately- and publicly-insured females than males (*p* < 0.01 and p < 0.01, respectively).

In the first sensitivity analysis with the extended HPV vaccine completion window to 18 months, 32.5% of females and 34.3% of males completed the 3-dose series. The pattern of associations between participant characteristics and HPV completion within 18 months were consistent with completion by 12 months (Supplemental Table 2). Similar to the main specification, the prevalence of vaccine completion fluctuated across the study period and adjusted trends showed significantly lower completion in 2013–2014 for females and males. Models using the extended completion window delivered similar results, with coefficients of a comparable magnitude to those from the 12-month model, confirming the robustness of our findings (Table 2).

In the second sensitivity analysis with the 2-dose schedule, 46.9% of females and 46.5% of males completed the series within 12 months. The pattern of associations between participant characteristics and HPV completion within 12 months were consistent (Supplemental Table 3). Similar to the main specification, both ACA provisions were associated with increases in HPV vaccine completion among females and privately-insured males (Table 2). In contrast, among publicly-insured males that received their first HPV vaccine, there was some evidence that the 2014 ACA reforms were associated with decreases in completion of the 2-dose series.

## Discussion

We have shown that despite low HPV vaccine completion overall, the 2010 ACA provision was associated with a modest increase in vaccine completion among females and the 2014 ACA-related health insurance reforms were associated with further increases in completion among females and males. While both privately- and publicly-insured youth benefited from the ACA, the 2014 insurance reforms had a larger effect among females than males. Furthermore, extending the HPV vaccine completion window from 12 to 18 months was associated with an increase in completion from approximately 28% to 33–34% in females and males. Despite minimal sex differences in HPV vaccine completion in these three New England states, publicly-insured youth continued to have lower completion than their privately-insured counterparts. Our findings suggest that Medicaid expansion in the 14 remaining states that have not yet expanded Medicaid [20] could increase HPV vaccine completion among publicly-insured youth and reduce their longer-term risk of HPV-related cancers [2].

State and national estimates of HPV vaccine initiation and completion are based on self-report of the number of HPV doses received without specifying a time frame for receipt [7, 15, 16]. Although the ACIP recommendation for completion of the 3-dose series at the time of this study required the third dose given at least 6 months after the first dose, this information is not recorded in surveys such as the NIS-Teen. A strength of population-based APCDs and claims-based studies [17, 21] are the use of objectively recorded medical claims with associated dates of service. Consistent with studies using private claims data [17, 21], we used a 12-month time frame for the 3-dose series completion from the month the first dose was received. We found an average prevalence of the HPV vaccine 3-dose series completion to be 30.5% among females and 27.5% among males in 2014, among those who initiated the HPV vaccine, with corresponding estimates of 69.3 and 57.8% from the NIS-Teen that same year [22]. Corroborating results by Spencer and colleagues [17], we found a higher prevalence of HPV vaccine completion by extending the vaccine window to 18 months—specifically, completion increased to 36.8% among females and 34.2% among males in 2014. This suggests that youth are often completing the required number of HPV doses well-beyond the recommended window. As the new 2016 ACIP guidelines requires only two doses for adolescents starting the series at ages 9–14 years [6], it will be important to monitor whether compliance increases due to fewer return visits. In a sensitivity analysis, we found overall consistent effects of both sets of ACA provisions on 2-dose HPV vaccine completion within 12 months except for publicly-insured males. However, as noted by Spencer and colleagues, it is challenging to use historical data to estimate future compliance as both the number of doses and timing of the second dose were modified [17].

This study also contributes to the literature in its inclusion of males. Prior evaluations of the 2010 ACA provision on HPV completion have been limited to females. Lipton and Decker found that the ACA increased HPV vaccine completion by 5.8 percentage points among women aged 19–25 years compared to 18- or 26-year-olds [15]. Corriero and colleagues also found that after the ACA was implemented, females aged 9–33 years were 5.8 times more likely to complete the 3-dose HPV series [16]. Despite the 2010 ACA provision only benefiting the privately-insured, neither study examined differential effects by insurance type [15, 16]. Consistent with these studies, we found that the ACA was associated with an increase in HPV vaccine completion among females; however, our results show that both privately- and publicly-insured females benefited from the policy—increasing vaccine completion by 4.3 and 5.7 percentage points, respectively. Although Lipton and Decker found no effect of the ACA on self-reported awareness of the HPV vaccine [15], increased insurance coverage and access to providers as a result of the ACA [23–25] may have resulted in an increased awareness of vaccination among providers for all young women.

This is one of the first studies to evaluate the 2014 ACA-related health insurance reforms on HPV vaccine completion. We found that the reforms were associated with an increase in vaccine completion among both privately- and publicly-insured females and males. For the privately-insured, state and federal Marketplaces offered publicly-subsidized private health insurance plans as well as the protections for those with pre-existing conditions. For the publicly-insured, Medicaid expansion due to the ACA provided preventive services with no cost sharing for those who qualified. Our findings demonstrate that increasing health insurance coverage, access to preventive services and reducing costs through the ACA directly benefited youth by increasing HPV vaccine completion rates.

In addition to the lack of insurance coverage and cost [10–12], having a regular medical provider, lack of provider recommendation, and being unaware or forgetting about additional doses have been identified as barriers specific to HPV vaccine completion [10]. Across these three New England states, youth who initiated the first vaccine at a younger age were more likely to complete the series, consistent with other studies [17, 21, 26]. We also found that privately-insured youth had higher HPV vaccine completion than Medicaid recipients. In contrast, the NIS-Teen has reported that publicly-insured adolescents were more likely to initiate and complete the HPV series than their privately-insured counterparts [7]. Based on the NIS-Teen, New England has the highest prevalence of HPV vaccine series completion (63.3%) across all regions and the US overall (48.6%) [7]. This may be due to differences in state programs available for Medicaid recipients or social norms related to HPV vaccination that differ regionally.

### Limitations

There are a number of limitations to note. APCDs only record insured enrollees, so uninsured youth, recognized to have the lowest uptake of HPV vaccine initiation and completion [7], were not included. Due to increases in health insurance coverage as a result of the ACA [14], the composition of the insured population in the APCDs also likely changed over the study period to include those with continuous coverage as well as the newly-insured. Since we conducted an individual-level analysis, our results suggest the association is less due to a change in the insured population because everyone in the analytic sample had at least one HPV vaccine dose. However, we cannot rule out the possibility that a newly-insured population may be more likely to complete the HPV vaccine series. Despite known racial/ethnic disparities in HPV vaccine initiation and completion [27, 28], APCDs also do not consistently collect information on race/ethnicity. There are other factors associated with HPV vaccine completion that we were not able to examine, including provider type or type of private insurance plan [17, 21, 26]; however, as these factors are more likely to be a result of ACA implementation, i.e. on the causal pathway, rather than moderate the relationship, they are not likely to confound the associations between implementation of the ACA provisions and HPV vaccine completion. Youth may have received the HPV vaccines through the Vaccines for Children Program, a program providing free vaccines for 18-year-olds and younger who are uninsured, underinsured, eligible for Medicaid, or American Indian or Alaskan Native [29]. Vaccines received through the Program or youth who paid for the vaccines out-of-pocket were not recorded in the APCD and may under-estimate the true prevalence of vaccine completion. The study only included data from three New England states, which may not be generalizable to the entire US. However, despite high levels of HPV vaccine completion in these states [7, 22], we still found robust associations between ACA policy changes and completion. This suggests our effect sizes may under-estimate the full impact of the ACA policy changes on HPV vaccine completion and regions in the US with a lower prevalence of completion may experience the same or larger gains in response to the ACA provisions. Finally, it is possible that other policy factors that occurred in the same time frame as the ACA policy changes were associated with HPV vaccine completion. We conducted two placebo tests by restricting the dataset prior to 2014 to evaluate the 2010 ACA policy and, separately, after 2010 to evaluate the 2014 ACA policy. There were similar effect sizes to the original combined model suggesting the robustness of the policy breaks (results not shown).

## Conclusions

Among cancers attributable to HPV in the US, 92% are associated with HPV types targeted by the 9-valent HPV vaccine [1]. Using APCDs from these three states, we previously found that the 2010 and 2014 ACA provisions increased HPV vaccine initiation rates among males and Medicaid recipients, but females and youth with private insurance did not exhibit these same increases in HPV vaccine uptake [18]. Among youth that initiated the HPV vaccine, we have shown that implementation of both sets of ACA provisions were associated with increases in completion of the series independent of sex and with similar gains among privately- and publicly-insured youth. Thus, expanding Medicaid across the remaining states [20] could further increase HPV vaccine initiation and completion as well as prevent HPV-related cancers [2] among this population.

## Supplementary Information

**Additional file 1 Table S1.** Average completion of the HPV vaccine series stratified by state, sex, and insurance type across study years. **Table S2.** Descriptive statistics for HPV vaccine completion defined as completion of the 3-dose series within 18 months. **Table S3.** Descriptive statistics for HPV vaccine completion defined as completion of the 2-dose series within 12 months.

## Data Availability

The All Payer Claims Databases that support the findings of this study are available from the Maine Health Data Organization (https://mhdo.maine.gov/), New Hampshire Comprehensive Health Care Information System (https://nhchis.com/), and Massachusetts Center for Health Information and Analysis (http://www.chiamass.gov/). Restrictions apply to the availability of these data, which were used under license for the current study, and so are not publicly available.

## Abbreviations

ACIP: Advisory Committee on Immunization Practices
APCD: All Payer Claims Databases
CI: confidence interval
HPV: human papillomavirus
MA: Massachusetts
ME: Maine
NH: New Hampshire
ACA: Patient Protection and Affordable Care Act
