# Supplementary material for: Associations between insurance-related affordable care act policy changes with HPV vaccine completion
Source: BMC Public Health. 2021 Feb 6;21:304. doi: 10.1186/s12889-021-10328-4 (PMC7866643; doi:10.1186/s12889-021-10328-4)
Supplement: Supplementary file 1 — Additional file 1 Table S1. Average completion of the HPV vaccine series stratified by state, sex, and insurance type across study years. Table S2. Descriptive statistics for HPV vaccine completion defined as completion of the 3-dose series within 18 months. Table S3. Descriptive statistics for HPV vaccine completion defined as completion of the 2-dose series within 12 months. [file 12889_2021_10328_MOESM1_ESM.docx]

**Supplemental Table 1** Average completion of the HPV vaccine series stratified by state, sex, and insurance type across study years

|  | Females | | | Males | | |
| --- | --- | --- | --- | --- | --- | --- |
|  | Massachusetts  % | Maine  % | New Hampshire % | Massachusetts  % | Maine  % | New Hampshire % |
| Private |  |  |  |  |  |  |
| 2009^a^ | - | 36.8 | 11.5 | - | - | - |
| 2010 | - | 42.4 | 23.0 | - | - | - |
| 2011 | 32.1 | 21.3 | 26.5 | 38.9 | 10.7 | 27.3 |
| 2012 | 38.8 | 23.4 | 23.2 | 40.3 | 22.7 | 24.2 |
| 2013 | 22.5 | 18.1 | 28.8 | 21.5 | 14.8 | 27.6 |
| 2014^a^ | 34.7 | 20.9 | 29.9 | 30.8 | 19.0 | 26.8 |
| Public |  |  |  |  |  |  |
| 2009^a^ | - | 6.1 | - | - | - | - |
| 2010 | - | 14.3 | 17.4 | - | - | - |
| 2011 | 15.1 | 18.2 | 24.0 | 17.8 | 19.4 | 32.4 |
| 2012 | 17.6 | 21.4 | 15.4 | 19.3 | 24.6 | 12.9 |
| 2013 | 16.6 | 17.5 | 10.7 | 17.3 | 16.5 | 11.2 |
| 2014^a^ | 21.8 | 18.0 | 11.3 | 18.0 | 17.8 | 12.7 |

^a^ Partial study year

**Supplemental Table 2** Descriptive statistics for HPV vaccine completion defined as completion of the 3-dose series within 18 months

|  | Females | | | | | Males | | | | |
| --- | --- | --- | --- | --- | --- | --- | --- | --- | --- | --- |
|  | N | % | % series completed | Unadjusted OR  (95% CI) | Adjusted OR (95% CI) | N | % | % series completed | Unadjusted OR  (95% CI) | Adjusted OR (95% CI) |
| Age of first dose (years) |  |  |  |  |  |  |  |  |  |  |
| 9-14 | 96702 | 49.7 | 37.2 | 1 | 1 | 72071 | 38.2 | 37.3 | 1 | 1 |
| 15-18 | 56407 | 29.0 | 31.6 | 0.78 (0.76-0.80) | 0.74 (0.73-0.76) | 87336 | 46.2 | 34.9 | 0.90 (0.88-0.92) | 0.82 (0.80-0.84) |
| 19-26 | 41298 | 21.2 | 22.9 | 0.50 (0.49-0.52) | 0.48 (0.46-0.49) | 29483 | 15.6 | 25.4 | 0.57 (0.56-0.59) | 0.51 (0.49-0.52) |
| Insurance type |  |  |  |  |  |  |  |  |  |  |
| Medicaid | 28827 | 14.8 | 22.1 | 1 | 1 | 22588 | 12.0 | 23.4 | 1 | 1 |
| Private | 165580 | 85.2 | 34.3 | 1.85 (1.79-1.90) | 2.58 (2.09-3.18) | 166302 | 88.0 | 35.8 | 1.83 (1.77-1.89) | 1.79 (1.73-1.86) |
| Year of first dose |  |  |  |  |  |  |  |  |  |  |
| 2009^a^ | 3565 | 1.8 | 23.8 | 1 | 1 | - | - | - | - | - |
| 2010 | 11397 | 5.9 | 31.5 | 1.47 (1.35-1.60) | 1.49 (1.36-1.63) | - | - | - | - | - |
| 2011 | 28463 | 14.6 | 30.7 | 1.42 (1.31-1.54) | 0.99 (0.88-1.13) | 6484 | 3.4 | 38.3 | 1 | 1 |
| 2012 | 59371 | 30.5 | 37.8 | 1.94 (1.80-2.10) | 1.24 (1.10-1.40) | 74231 | 39.3 | 41.8 | 1.16 (1.10-1.22) | 1.09 (1.03-1.15) |
| 2013 | 64134 | 33.0 | 27.2 | 1.19 (1.10-1.29) | 0.69 (0.61-0.78) | 78172 | 41.4 | 27.0 | 0.60 (0.57-0.63) | 0.52 (0.49-0.55) |
| 2014^a^ | 27477 | 14.1 | 36.8 | 1.86 (1.72-2.02) | 0.84 (0.74-0.96) | 30003 | 15.9 | 34.2 | 0.84 (0.79-0.89) | 0.59 (0.55-0.64) |
| State |  |  |  |  |  |  |  |  |  |  |
| Massachusetts | 136351 | 70.1 | 34.9 | 1 | 1 | 151877 | 80.4 | 36.5 | 1 | 1 |
| Maine | 24761 | 12.7 | 27.3 | 0.70 (0.68-0.72) | 0.66 (0.64-0.69) | 15160 | 8.0 | 22.6 | 0.51 (0.49-0.53) | 0.50 (0.48-0.52) |
| New Hampshire | 33295 | 17.1 | 26.4 | 0.67 (0.65-0.69) | 0.61 (0.59-0.63) | 21853 | 11.6 | 27.5 | 0.66 (0.64-0.68) | 0.63 (0.61-0.65) |

Abbreviations: CI, confidence interval; HPV, human papillomavirus

^a^ Partial study year

**Supplemental Table 3** Descriptive statistics for HPV vaccine completion defined as completion of the 2-dose series within 12 months

|  | Females | | | | | Males | | | | |
| --- | --- | --- | --- | --- | --- | --- | --- | --- | --- | --- |
|  | N | % | % series completed | Unadjusted OR  (95% CI) | Adjusted OR (95% CI) | N | % | % series completed | Unadjusted OR  (95% CI) | Adjusted OR (95% CI) |
| Age of first dose (years) |  |  |  |  |  |  |  |  |  |  |
| 9-14 | 96702 | 49.7 | 49.8 | 1 | 1 | 72071 | 38.2 | 48.2 | 1 | 1 |
| 15-18 | 56407 | 29.0 | 45.5 | 0.84 (0.83-0.86) | 0.82 (0.80-0.83) | 87336 | 46.2 | 47.0 | 0.95 (0.93-0.97) | 0.91 (0.89-0.93) |
| 19-26 | 41298 | 21.2 | 42.3 | 0.74 (0.72-0.76) | 0.74 (0.72-0.75) | 29483 | 15.6 | 40.9 | 0.74 (0.72-0.76) | 0.71 (0.69-0.73) |
| Insurance type |  |  |  |  |  |  |  |  |  |  |
| Medicaid | 28827 | 14.8 | 42.3 | 1 | 1 | 22588 | 12.0 | 38.0 | 1 | 1 |
| Private | 165580 | 85.2 | 48.6 | 1.60 (1.56-1.64) | 2.17 (1.84-2.56) | 166302 | 88.0 | 47.7 | 1.49 (1.44-1.53) | 1.79 (1.73-1.86) |
| Year of first dose |  |  |  |  |  |  |  |  |  |  |
| 2009^a^ | 3565 | 1.8 | 40.1 | 1 | 1 | - | - | - | - | - |
| 2010 | 11397 | 5.9 | 48.4 | 1.40 (1.30-1.51) | 1.42 (1.31-1.54) | - | - | - | - | - |
| 2011 | 28463 | 14.6 | 47.6 | 1.36 (1.26-1.45) | 1.20 (1.07-1.34) | 6484 | 3.4 | 47.5 | 1 | 1 |
| 2012 | 59371 | 30.5 | 50.3 | 1.51 (1.41-1.62) | 1.30 (1.17-1.46) | 74231 | 39.3 | 52.1 | 1.20 (1.14-1.26) | 1.18 (1.12-1.24) |
| 2013 | 64134 | 33.0 | 40.8 | 1.03 (0.96-1.10) | 0.84 (0.76-0.94) | 78172 | 41.4 | 39.6 | 0.72 (0.69-0.76) | 0.69 (0.65-0.72) |
| 2014^a^ | 27477 | 14.1 | 53.6 | 1.73 (1.61-1.85) | 1.51 (1.34-1.71) | 30003 | 15.9 | 50.5 | 1.13 (1.07-1.19) | 1.11 (1.03-1.19) |
| State |  |  |  |  |  |  |  |  |  |  |
| Massachusetts | 136351 | 70.1 | 47.3 | 1 | 1 | 151877 | 80.4 | 46.9 | 1 | 1 |
| Maine | 24761 | 12.7 | 48.3 | 1.04 (1.01-1.07) | 1.08 (1.05-1.11) | 15160 | 8.0 | 42.9 | 0.85 (0.82-0.88) | 0.89 (0.86-0.92) |
| New Hampshire | 33295 | 17.1 | 44.7 | 0.90 (0.88-0.92) | 0.88 (0.86-0.91) | 21853 | 11.6 | 46.6 | 0.99 (0.96-1.02) | 0.97 (0.95-1.00) |

Abbreviations: CI, confidence interval; HPV, human papillomavirus

^a^ Partial study year
